# The role of microtubules in microalgae: promotion of lipid accumulation and extraction

**DOI:** 10.1186/s13068-023-02257-8

**Published:** 2023-01-12

**Authors:** Lijie Zhang, Xiao Lin, Zhigang Yang, Liqun Jiang, Qingjie Hou, Zhen Xie, Yizhen Li, Haiyan Pei

**Affiliations:** 1 School of Environmental Science and Engineering, Shandong University, Qingdao, 266237 China; 2 Department of Environmental Science and Engineering, Fudan University, Shanghai, 200433 China; 3 Shandong Provincial Engineering Center on Environmental Science and Technology, Jinan, 250061 China; 4 Department of Chemical Engineering and Biotechnology, University of Cambridge, Cambridge, CB3 0AS UK

**Keywords:** Microalgae, Microtubule destruction, Two-stage cultivation, Lipid accumulation, Cell fragility

## Abstract

**Background:**

Microtubules in cells are closely related to the growth and metabolism of microalgae. To date, the study of microalgal microtubules has mainly concentrated on revealing the relationship between microtubule depolymerization and synthesis of precursors for flagellar regeneration. While information on the link between microtubule depolymerization and biosynthesis of precursors for complex organic matter (such as lipid, carbohydrate and protein), is still lacking, a better understanding of this could help to achieve a breakthrough in lipid regulation. With the aim of testing the assumption that microtubule disruption could regulate carbon precursors and redirect carbon flow to promote lipid accumulation, *Chlorella sorokiniana* SDEC-18 was pretreated with different concentrations of oryzalin.

**Results:**

Strikingly, microalgae that were pretreated with 1.5 mM oryzalin accumulated lipid contents of 41.06%, which was attributed to carbon redistribution induced by microtubule destruction. To promote the growth of microalgae, two-stage cultivation involving microtubule destruction was employed, which resulted in the lipid productivity being 1.44 times higher than that for microalgae with routine single-stage cultivation, as well as yielding a desirable biodiesel quality following from increases in monounsaturated fatty acid (MUFA) content. Furthermore, full extraction of lipid was achieved after only a single extraction step, because microtubule destruction caused removal of cellulose synthase and thereby blocked cellulose biosynthesis.

**Conclusions:**

This study provides an important advance towards observation of microtubules in microalgae through immunocolloidal gold techniques combined with TEM. Moreover, the observation of efficient lipid accumulation and increased cell fragility engendered by microtubule destruction has expanded our knowledge of metabolic regulation by microtubules. Finally, two-stage cultivation involving microtubule destruction has established ideal growth, coupling enhanced lipid accumulation and efficient oil extraction; thus gaining advances in both applied and fundamental research in algal biodiesel production.

**Supplementary Information:**

The online version contains supplementary material available at 10.1186/s13068-023-02257-8.

## Introduction

As a sustainable energy resource, microalgae have become hotspot in bioenergy research in these years, with the potential to be a viable alternative to fossil fuels, spurred by the advantageous characteristics of high growth rates and lipid productivity, carbon-neutrality, and the capacity to respond to external stimuli [1–4]. It is noteworthy that microtubules, forming a plant’s cytoskeleton, play pivotal roles in cell division and proliferation, intracellular and intercellular transport, cellular organization, and the changes in cell wall structural components [5–7]. A systematic analysis of microtubular changes, which could regulate microalgal metabolism, may bring breakthroughs for stimulation of lipid production in microalgae.

Previous studies have confirmed the contributions of microtubules to higher plant fitness under environmental stimuli, such as in *Arabidopsis*, tomatoes, tobacco, etc*.* [8–10]. Furthermore, recent studies provide important insights into microtubules at the genetic level, describing the molecular details of microtubule dynamics [7, 11, 12]. Notwithstanding the extensive efforts devoted to microtubules in plants, those studies still only focused on higher plants, mainly including *Arabidopsis*, wheat, rice, etc. Thus, little information has been available concerning microtubules in microalgae, due to the difficulty of observation. To bridge this gap, exploration of the relationships between microtubules and flagellar regeneration in a model alga, *Chlamydomonas reinhardtii*, can be an important step forward for microtubule study in microalgae. To date, the study of microalgal microtubules has mainly concentrated on the target of *Chlamydomonas reinhardtii* and revealing the relationship between microtubule depolymerization and synthesis of precursors for flagellar regeneration [13–15]. While information on the link between microtubule depolymerization and biosynthesis of precursors for complex organic matter (such as lipid, carbohydrate and protein), is still lacking, a better understanding of this could help to achieve a breakthrough in lipid regulation.

Microtubules are filamentous subcellular structures mainly composed of the tubulins and heterodimeric protein [16]. In the higher plant cells, microtubules organize into cortical networks tethered to the plasma membrane (PM), which serve as tracks for PM-localized cellulose synthase complexes (CSCs) and thereby determine cellulose deposition. As elucidated in the investigation of Wang et al., Kumar et al. and Motta et al. [7, 17, 18], if the microtubules in a higher plant were disrupted, the microtubule-guided cellulose synthase would be removed from the membrane, which could further affect the synthesis of cellulose. Therefore, whether microtubule destruction in microalgae could affect cellulose synthesis is an important question worthy of study. As such, the question is what will happen to carbon allocation in microalgae with the disruption of microtubule proteins and blocking of cellulose synthesis? Also important are the issues of whether microtubules could affect carbon allocation, and whether microalgae could redirect carbon flow towards lipid formation when microtubular protein and cellulose synthesis is blocked. Therefore, a better understanding of those matters would achieve a biotechnology breakthrough in lipid induction to expand knowledge of metabolic regulation by microtubules in microalgae. Strikingly, microtubule disruption would also make microalgal cells fragile because cellulose, synthesized by microtubule-guided cellulose synthase, provides toughness to the cell wall [17, 19], and so that would ease the bottleneck of lipid extraction.

Nevertheless, under conditions of microtubule disruption, growth inhibition has become the major concern, as a lower biomass would lead to an undesirable lipid productivity. To overcome the challenge two-stage cultivation is proposed, in which microalgae would grow under ideal conditions in the first stage, and then the microtubules would be destroyed, allowing accumulation of lipid, when entering the stationary phase. This two-stage cultivation involving microtubule destruction could establish the combination of ideal growth, enhanced lipid accumulation and efficient oil extraction; thus providing an important advance towards microalga-based biofuel production.

Within these contexts, we chose *Chlorella sorokiniana* SDEC-18 as the subject algae and oryzalin as the microtubule depolymerizing agent. The main aims in our study were: (1) to understand the effects of microtubules on cell division and elongation in microalgae; (2) to track microalgal lipid, microtubule protein and cellulose accumulation under different degrees of microtubule depolymerization as functions of cultivation time; (3) to test whether microtubule disruption could make microalgal cells fragile, and hence ease the bottleneck of lipid extraction; and (4) to propose and demonstrate a two-stage-cultivation strategy involving microtubule destruction to accelerate the commercialization of microalgal biofuel production.

## Materials and methods

### Algal species

*Chlorella sorokiniana* SDEC-18 was isolated and screened by our research group from Quancheng Lake in Jinan, which was pre-cultured in BG11 medium [20].

### Experimental design

To understand the effects of microtubules on cell growth and lipid accumulation in microalgae and to select the optimum depolymerization concentration, the same number of cells, pretreated by depolymerization of 30%, 60% and 100% of the microtubules, corresponding to 0.5 mM, 1.5 mM and 3 mM oryzalin, were grown in normal medium over a 10-day period (Additional file 1: Fig. S1a). Overall 1.5 mM was the best oryzalin concentration for depolymerization, with ideal growth and lipid accumulation.

Two-stage cultivation was conducted after the optimal depolymerization concentration was selected. The healthy microalgae were pre-grown in 1-L Erlenmeyer flasks for a period of 6 days, with entry into the stationary phase. After that, approximately 40 mL samples of dense algal cells at the bottom were collected by sedimentation, and then 1.5 mM of oryzalin was added, which ensured the recycling of BG11 and reduced the excessive use of oryzalin (Additional file 1: Fig. S1b).

### Analytical methods

#### Indicators of microalgal growth

##### Biomass accumulation

Microalgal growth was monitored after every 24 h by measuring the absorbance at 680 nm. The dry mass (DM) of the algae was calculated following the equation:

$$ {\text{DM }} = \, 0.{\text{657 OD}}_{{{68}0}} {-} \, 0.0{841}{\text{.}} $$

##### Chlorophyll a accumulation

Pigment contents were calculated as described by Zhang et al. [21]. About 2 mL of centrifuged algal culture were resuspended in methanol, which were incubated for 24 h at 45 °C in the dark and then centrifuged, and the absorbances of the extracted supernatant at 652.4 and 665.2 nm were determined by UV–visible spectrophotometer:

$$ {\text{Chlorophyll a}}: {\text{Chl}} - {\text{a }}\left( {{\text{mg}}/{\text{L}}} \right) \, = { 16}.{72}A_{{{665}.{2}}} {-}{ 9}.{16}A_{{{652}.{4}}} . $$

##### Cell number and size

Microalgae cell morphology, numbers and size were observed with an optical microscope (CX31, Olympus, Japan) and inverted fluorescence microscope (Ti-s, Nikon, Japan), respectively.

#### Lipid accumulation

##### Lipid content

The lipid was extracted from the dried microalgal biomass using chloroform:methanol (2:1, v/v), and determined gravimetrically and expressed on a dry weight basis as described by Zhang et al*.* [20]. The lipid extractions were carried out several times until the lipid was completely extracted. The lipid productivity was determined by dry mass, lipid content and cultivation time:

$$  {\text{LC }} = {\mkern 1mu} \left( {m_{2}  - m_{0} } \right){\mkern 1mu}  \times V/5 \times m_{1} ,  $$

where LC, *m*_0_, *m*_1_, *m*_2_ and *V* represent lipid content (%), dry mass of glass tubes, dry mass of algal pellets, dry mass of glass tubes with lipid, and volume of low temperature grease.

##### Fluorescence images of Nile red-stained neutral lipid

Firstly, about 750 μL of concentrated microalgae samples was mixed with 250 μL dimethyl sulfoxide (DMSO), subsequently 20 μL of Nile red dye was added and incubated for 10 min in the dark, and then the observation of neutral lipid was performed with inverted fluorescence microscope as described in Zhang et al. [20].

##### Lipid droplet observation through transmission electron microscopy

2.5% glutaraldehyde was used to fix microalgae cells after centrifugation, and then, after routine immersion, 1% osmium acid fixation, phosphate buffer rinsing, gradient dehydration, embedding and other steps, semi-thin section positioning was performed to prepare ultra-thin section samples of microalgae. After electron staining with uranyl acetate and lead citrate, it was observed with JEOL-1200EX transmission electron microscope.

#### Microtubules

##### Microtubule content

Microtubule content was determined as depicted by Parreno et al. [22] and Liang et al. [23]. Samples of algae were harvested by centrifuging at 5000 r/min for 10 min, and the pellets were treated with 5 mL of 1% Triton X-100 for 20 min to extract the proteins other than microtubule protein. The algal samples were then centrifuged at 5000 r/min for 10 min, and the supernatant was discarded. After that the pellets were resuspended with 10 mL of distilled water and microalgal samples were treated by ultrasound in an ice bath with an ultrasonic Cell Crusher (SCIENTZ-IID, China) for 10 min, and then immediately centrifuged at 5000 r/min for 10 min. A total of 0.1 mL of supernatant, 0.9 mL of distilled water and 5 mL of Coomassie brilliant blue G-250 reagent were mixed, and the mixture’s absorbance at 595 nm was recorded. The protein content was calculated from a standard calibration curve.

##### Observation of microtubules’ fluorescence

Microtubules in the microalgae were observed using a high-sensitivity laser confocal microscope, LSM 780, according to the protocol described by Park et al*.* [12] and Sugimoto et al*.* [24]. The primary antibody was a mouse monoclonal antibody against β-tubulin (Sigma, USA) at 1:150 dilution and the secondary antibody was fluorescein isothiocyanate-conjugated anti-mouse IgG (Silenus/Amrad Biotech, Melbourne, Australia) at 1:400 dilution. The excitation wavelength of the samples was 488 nm, accordingly, the emission wavelength was 505–530 nm.

##### Microtubule observation through transmission electron microscopy

According to the traditional transmission electron microscope sample preparation method, semi-thin section positioning was performed to prepare ultra-thin section samples of microalgae. Ultrathin sections were collected on a nickel screen for preparation of colloidal gold markers. The microtubules were observed using a JEOL-1200EX transmission electron microscope (JEOL, Akishima, Japan) as described by Danilov et al. [25]. The primary antibody was a mouse monoclonal antibody against β-tubulin (Sigma, USA) at 1:150 dilution and the secondary antibody was Au-Goat anti-mouse IgG (H + L) (Sigma, USA) at 1:50 dilution.

#### Cellulose

##### Cellulose content

According to Viles et al*.* [26], about 0.1 g dried algae powder was mixed with 60% H_2_SO_4_ and digested for 30 min in a cold bath, which was then diluted to 100 mL with 60% H_2_SO_4_ to obtain the cellulose extract. And a Multiskan FC (Thermo, USA) was applied to the detection of cellulose content at a 620 nm wavelength.

##### Measurement of cellulose synthase content

The cellulose synthase content was measured using Cellulose synthase (CEsA) enzyme-linked immunosorbent assay kit (Shanghai Kanglang Biological Technology Co., Ltd.) according to the instruction manual.

#### Determination of carbon redistribution in microalgae

##### Total lipid content and fatty acid methyl ester (FAME)

The total lipid content was determined in accordance with the above 2.3.2.1. And the fatty acid methyl ester (FAME) was extracted using the one-step transesterification method, and then identified and quantified by gas chromatography–mass spectrometry (GC–MS) (Trace GC-DSQII, Thermo Fisher, USA) as described by Zhang et al. [27].

##### Total carbohydrate content

The total carbohydrate was determined by the dinitrosalicylic acid method at a wavelength of 620 nm as described by Zhang et al. [27].

##### Total protein content

As described in Zhang et al*.* [28], the total protein content was determined by the Coomassie Brilliant Blue G-250 method at a wavelength of 595 nm.

#### Lipid-extraction efficiency

##### Algal cell disruption

The algal cell fragility was characterized by cell disruption. Microalgal samples were treated by ultrasound with an ultrasonic Cell Crusher (SCIENTZ-IID, China) for 30 min, which were then observed under an optical microscope (CX31, Olympus, Japan), and the percentage of disrupted cells was obtained from the formula:

$$ \% {\text{disruption }} = N_{{{\text{disrupted}}}} /N_{{{\text{all}}}} \times { 1}00\% , $$

where *N*_disrupted_ and *N*_all_ represent the number of disrupted algal cells after ultrasound and all algal cells, respectively.

##### Lipid extractability

The lipid was extracted from the dried microalgal biomass using chloroform:methanol (2:1, v/v), and the lipid extractions were carried out several times until the lipid was completely extracted.

#### Statistical analysis

All experiments were performed in triplicate and the results were evaluated by analysis of variance (ANOVA) with the level of significance (*p* < 0.05).

## Results and discussion

### Enhancement of microalgal lipid accumulation and extraction through microtubule disruption

Bioprospecting for microalgae that undergo microtubule disruption capable of accumulating lipid droplets can clear a path for scales of microalgae biodiesel production. Thus, to gain mechanistic insights into lipid accumulation within microalgae subject to microtubule destruction, it is a wise choice to use oryzalin as a microtubule depolymerizing agent, as this would expand the life connotation of microtubules and lipid accumulation in algae cells.

**Fig. 1:**
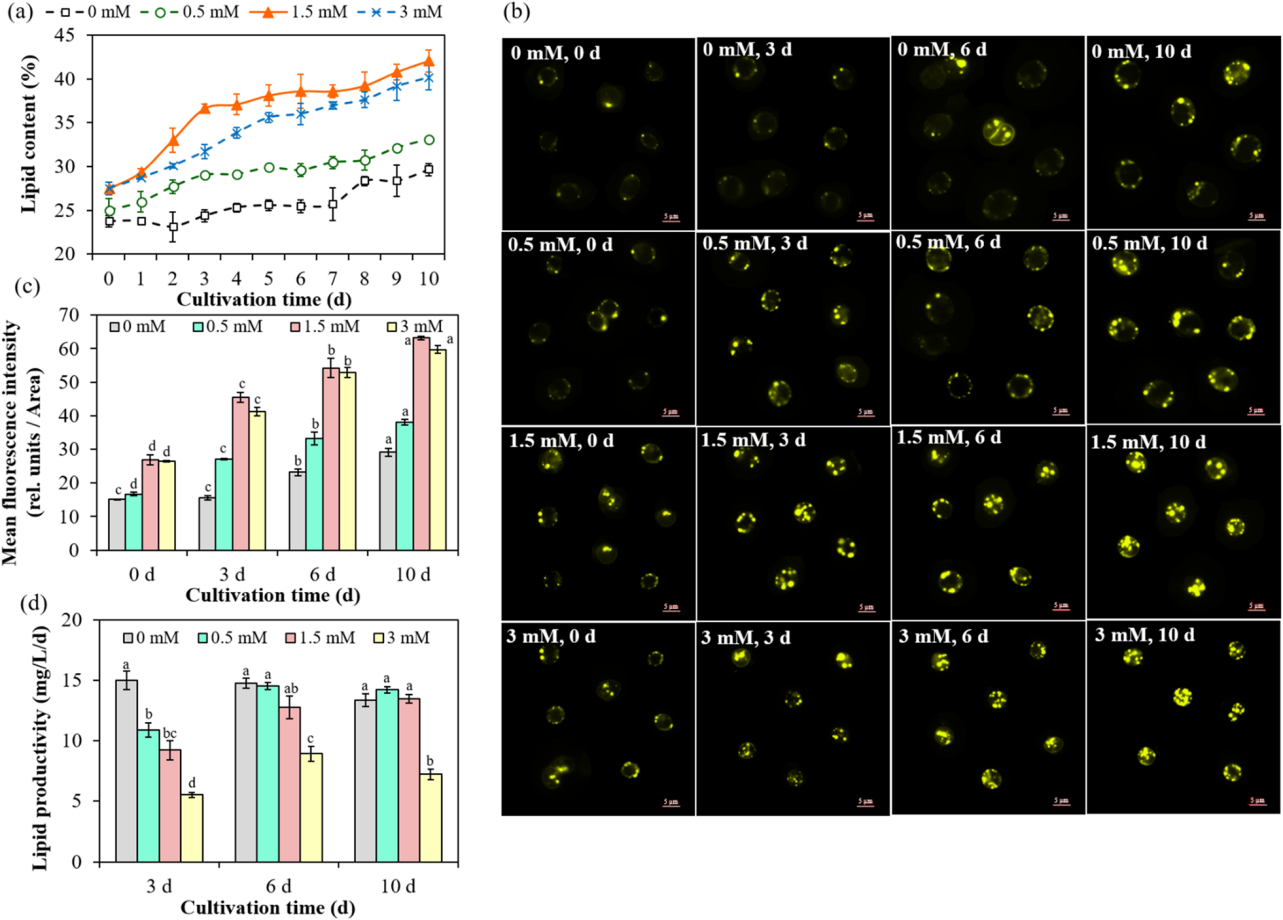
Lipid accumulation in *Chlorella sorokiniana* SDEC-18 pretreated with 0 mM, 0.5 mM, 1.5 mM and 3 mM of oryzalin: **a** lipid content as a function of cultivation time; **b** Nile red-stained neutral lipid within microalgal cells; **c** fluorescence intensity of Nile red-stained microalgal cells at 0, 3, 6 and 10 d; and **d** lipid productivity as a function of cultivation time. Results for a given treatment that are not annotated with the same letter demonstrated a statistically significant difference (*p* < 0.05) between the corresponding cultivation times and corresponding treatments

With curiosity, *Chlorella sorokiniana* SDEC-18 pretreated to depolymerize 30%, 60% and 100% of microtubules (corresponding to 0.5 mM, 1.5 mM and 3 mM oryzalin) were grown in normal medium over a 10-day period. As plotted in Fig. 1a, an increase in lipid content was recorded in microalgal cells pretreated with oryzalin as compared to microalgal cells containing complete microtubules. *Chlorella sorokiniana* SDEC-18 pretreated with oryzalin accumulated lipid rapidly during early-stage culture, especially for microalgae pretreated with 1.5 mM oryzalin, with depolymerization of 60% of the microtubules: the lipid content sharply increased from 27.48% (Day 0) to 36.62% (Day 3) and kept increasing slowly thereafter, attaining a final lipid content of 41.06%. Compared to the response in 1.5 mM oryzalin, the growth trends in lipid content for *Chlorella sorokiniana* SDEC-18 pretreated with 0.5 mM and 3 mM oryzalin (with depolymerization of 30% and 100% of microtubules, respectively) were considerably moderated. Whereas for untreated microalgae (0 mM) the lipid content remained constant during early-stage culture (Day 0 to Day 7), and increased slowly only during the stationary period.

Fluorescence microscopy provides images for morphological analysis, including the subcellular patterns of live cells. Generally lipid content could be presented qualitatively in the form of fluorescence stained with Nile red [29]. As shown in Fig. 1b, consistent with the lipid content, fluorescence always remained weak until a slight fluorescence emerged on the last day in untreated microalgae (0 mM). Strikingly, a relatively bright fluorescence in microalgae pretreated with oryzalin appeared from the start when transferred to normal medium, indicating microalgae began to accumulate lipid during pretreatment, and continued to markedly increase up to the last day—the mean fluorescence intensity of lipid droplets reaching 38.02, 63.24 and 59.75 rel. units/Area in 0.5, 1.5 and 3 mM oryzalin (respectively)—being 1.30, 2.17 and 2.05 times higher than in untreated microalgae (0 mM) (Fig. 1c). This further confirmed that microtubule depolymerization can indeed promote the accumulation of lipid, and 1.5 mM was the best oryzalin concentration for depolymerization.

The lipid distribution in microalgal cells could also be observed through transmission electron microscopy (TEM) (Additional file 1: Fig. S2), supporting our content measurement results. In untreated medium (0 mM), only electron-sparse punctuations in lipid bodies occurred in microalgae containing complete microtubules, while for microalgae pretreated with oryzalin the mass of lipid bodies gradually filled a large proportion of the cell space by the 3^rd^ day. These observations consolidated the view that microtubule destruction did indeed promote lipid accumulation in microalgae cells.

Noteworthily, the lipid productivity of *Chlorella sorokiniana* SDEC-18 pretreated with 1.5 and 0.5 mM oryzalin did not show a noticeable difference from the productivity in untreated microalgae (0 mM) at last (Fig. 1d). That was because the lipid enhancement compensated for the growth inhibition due to microtubule depolymerization [5, 6], which further confirmed although microtubule depolymerization affected microalgae growth, it did not reduce the lipid productivity of microalgae because it promoted the accumulation of intracellular oil.

**Fig. 2:**
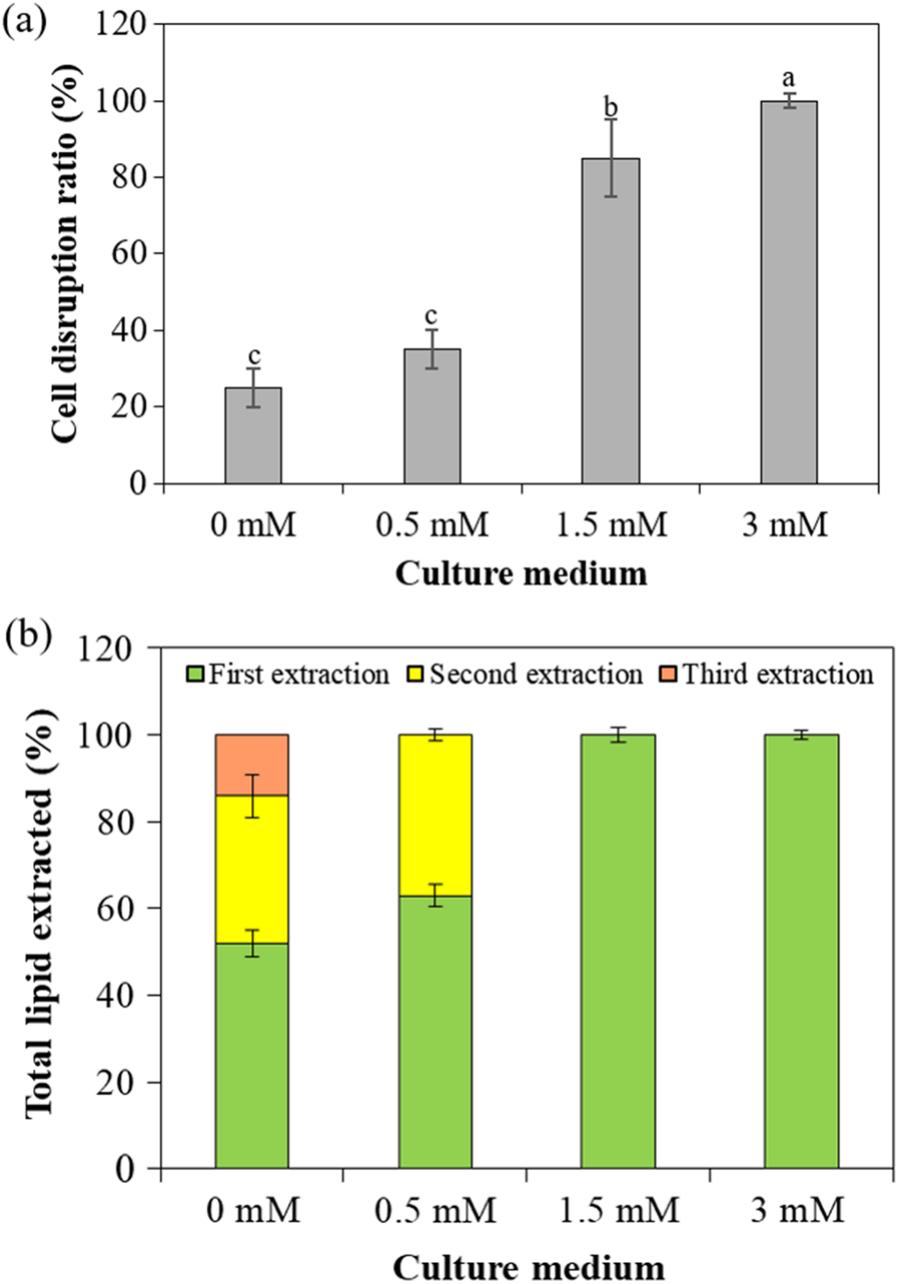
The microalgal cell disruption and lipid-extraction efficiency of *Chlorella sorokiniana* SDEC-18 pretreated with 0 mM, 0.5 mM, 1.5 mM and 3 mM of oryzalin. **a** cell disruption ratio using 30 min of sonication; **b** total lipid extracted of different extraction times. Results not annotated with the same letter demonstrated a statistically significant difference (*p* < 0.05) between the corresponding treatments

Microtubule disruption was able to not only stimulate lipid accumulation, but also promote lipid extraction. As shown in Fig. 2a, the microalgal cells were substantially disrupted using 30 min of sonication, while, interestingly, the cell disruption ratio was only able to reach 25% for microalgae without pretreatment. By way of comparison, the algal cell disruption fractions for microalgae that experienced microtubule destruction corresponding to 0.5, 1.5 and 3 mM oryzalin pretreatment were able to reach 35%, 85% and 100%, which were up to 3–4 times higher than that obtained in 0 mM medium. Perhaps unsurprisingly then, microtubule destruction altered structural integrity and cell wall components to make cells fragile, and thus rendering it easier to extract lipids from cells.

As depicted in Fig. 2b, following the single sonication treatment and first organic solvent extraction, 52% lipid extraction was obtained in microalgae without pretreatment of oryzalin. Extraction of nearly 100% of the lipid required 3 successive extractions, with an energy expense of sonication exceeding 47,500 kWh/kg and the added cost of the organic solvent. In contrast, for microalgae in 1.5 and 3 mM oryzalin-pretreatment media, complete lipid extraction was achieved after only one extraction step due to the easier cell disruption. Therefore, lipid extraction from microalgae pretreated with oryzalin is much less energy intensive.

### Causes of enhancement of lipid accumulation and extraction for microalgae subjected to microtubule disruption

#### The destabilization of microtubules

**Fig. 3:**
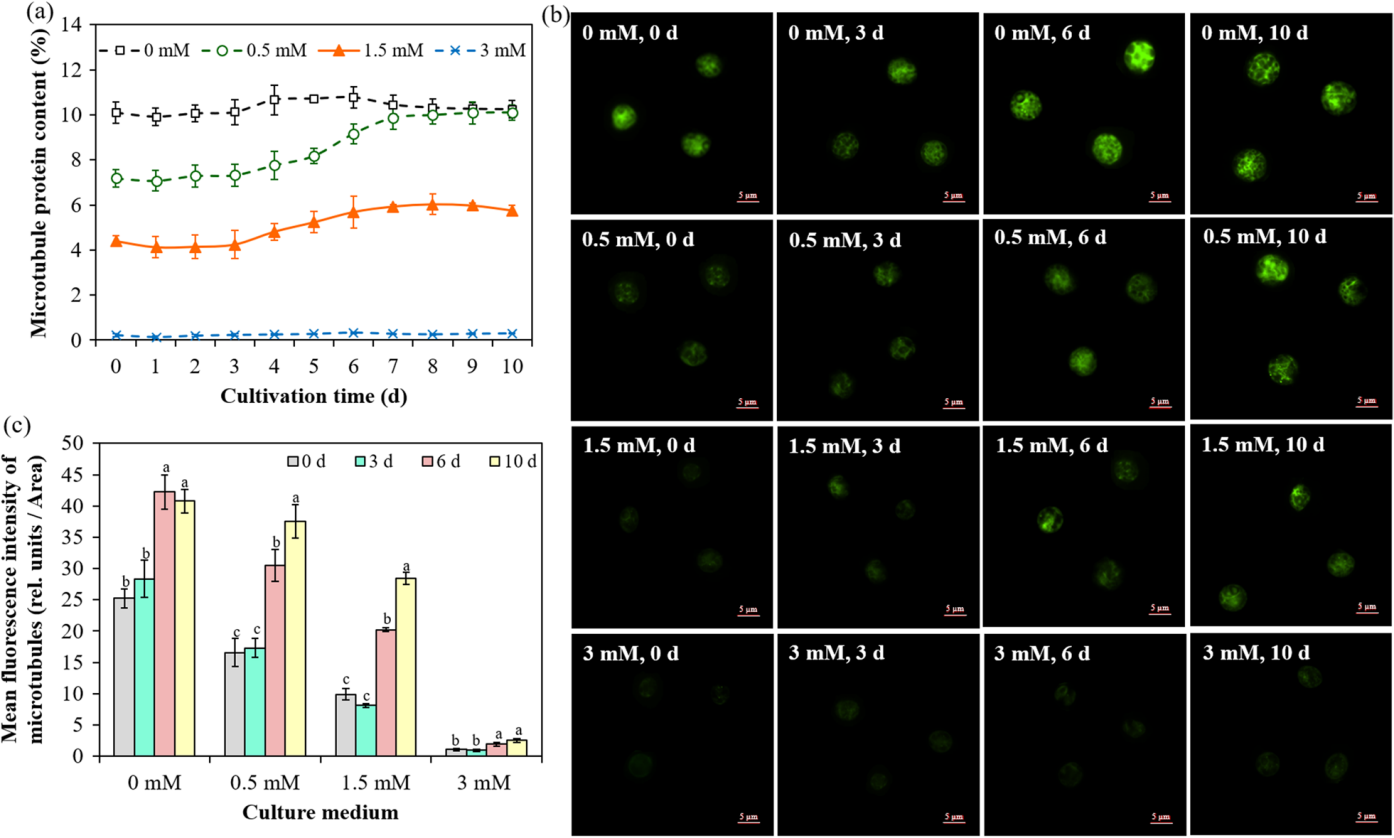
Microtubule changes within *Chlorella sorokiniana* SDEC-18 pretreated with 0 mM, 0.5 mM, 1.5 mM and 3 mM of oryzalin: **a** changes in microtubule protein content with cultivation time; **b** fluorescence micrographs showing microtubules within microalgae at 0, 3, 6 and 10 d; and **c** fluorescence intensity of microtubules within microalgae at 0, 3, 6 and 10 d. Results for a given treatment not annotated with the same letter demonstrated a statistically significant difference (*p* < 0.05) between the corresponding cultivation times

The time-course profiles of microtubule protein content in microalgae pretreated with different concentrations of oryzalin are presented in Fig. 3a. For *Chlorella sorokiniana* SDEC-18 without pretreatment (0 mM), the microtubule protein content was in dynamic balance, which was because microtubules were always in dynamic changes of assembly and disassembly, and that was also the reason and basis for plants to exhibit multiple structures and perform multiple functions [30, 31]. With respect to *Chlorella sorokiniana* SDEC-18 pretreated with 0.5 and 1.5 mM oryzalin, the microtubule protein remained low at early times (Day 0 to Day 3), due to microtubule disruption, and increased slowly thereafter, which follows from the suggestion that microtubules can be used as sensors to respond to external environmental stress [32]. When faced with an inhospitable environment, such as hyperosmotic stress or the presence of microtubule depolymerizing agent, microtubules could disassemble rapidly to respond [30, 33]. After a prolonged time in an inhospitable environment, the microtubule array gradually re-emerged, possibly due to the gradual adaptation to the inhospitable environment [34, 35]. However, as regards microalgae pretreated with 3 mM oryzalin, the microtubule protein was not resynthesized, because if the microtubules were destroyed so severely, they would be insufficiently stable even for reassembly [36]. With respect to microalgae pretreated with 0.5 or 1.5 mM oryzalin, the microtubule protein content remained low and then increased after 3 days’ cultivation; in the meantime, the accumulation rate of lipid also tended to be slow from the 3rd day. In comparison, following pretreatment with 3 mM oryzalin the microtubule protein content remained low throughout the period, while in parallel the lipid content kept rising during the whole process. Yet for microalgae without pretreatment (0 mM) the contents of microtubule protein and lipid had little changes throughout the entire cultivation period. Therefore, the above conjecture could be confirmed—microtubules did regulate the lipid synthesis—due to the observation that the reversals in microtubule protein and lipid contents occurred at nearly the same time.

As shown in Fig. 3b, consistent with the determination results of the above-mentioned microtubule protein content, the microtubule regions characterized by green fluorescence remained brightly colored throughout the cultivation period in the microalgae without pretreatment (0 mM), and the bright fluorescence was maintained at about 40.79 rel. units/Area (Fig. 3c). As for microalgae pretreated with 0.5 and 1.5 mM of oryzalin, the microtubule regions were dim for the first several days, due to the microtubules’ disruption by the pretreatments, and then became brighter, with fluorescence intensities of 37.54 and 28.44 rel. units/Area on the final day. For microalgae pretreated with 3 mM oryzalin, consistent with the measurements of microtubule protein content in Fig. 3a, the microtubule regions remained dark during the whole process, because the microtubule protein was not resynthesized [36], and the fluorescence intensity of microtubule regions was only 2.56 rel. units/Area.

The immunogold labeling technique combined with transmission electron microscopy (TEM) further advanced the localization of microtubule components to the submicroscopic level, which is an important supplement to the results obtained by immunofluorescence technology. The immunogold particles possess high electron density characteristics, have high resolution under an electron microscope, less coverage on ultrastructures, and are easy to distinguish from other granular structures so as to have more accurate positioning ability [25]. It is essential to use immunocolloidal gold techniques combined with TEM to reveal the distribution and changes of microtubules in the cells, which is helpful for studying the structure, function and regulation mechanisms in microalgal cells. As observed in immunogold electron micrographs, dense distribution of colloidal gold existed in algae cells containing intact microtubules (Additional file 1: Fig. S3), whereas for the microalgae pretreated with oryzalin only a sparse distribution of colloidal gold was recorded in microalgal cells, signifying incomplete microtubules. Endler et al*.* have provided insights on badly damaged microtubules, which were not stable, and the cellulose synthase cannot return to the cell membrane to fill the plasma membrane, causing morphological defects of algal cells [19]. As presented in Additional file 1: Fig. S3, microalgae that experienced microtubule destruction underwent severe deformation, and microtubule destruction was able to cause separation between the cell plasma and wall, that was, physical change at the interface of the plasma membrane and cell wall.

Through these microtubule protein/lipid content measurements and observations with staining and TEM we confirmed that microtubules are able to regulate lipid synthesis. Furthermore, a systematic analysis of the mechanisms lying behind the effect of microtubule proteins’ regulation on lipid synthesis is an urgent need, a better understanding of which would achieve a biotechnology breakthrough in lipid induction to expand knowledge of metabolic regulation by microtubules in microalgae.

#### The reduction of cellulose synthesis

The unfavorable environment could change the cell wall composition by regulating the synthesis of related proteins [36]. The cellulose, embedded in a hydrated matrix of hemicellulose, pectin, and glycoproteins, is the main component of the cell wall, which provides rigidity to the cell wall [17, 36, 37]. The synthesis of cellulose is mainly attributed to the catalytic integration of cellulose synthase which is mainly fixed and directed on the cell membrane by microtubules [38–40]. It has been reported that microtubule could respond quickly to the external environment and rapidly depolymerize, which weakened cell wall through reducing cellulose deposition [33, 41–44]. In addition, microtubules can determine the strength of the cell wall, and the dynamics of microtubules also depend on the mechanical properties of the cell wall, the two of which are interdependent [32].

**Fig. 4:**
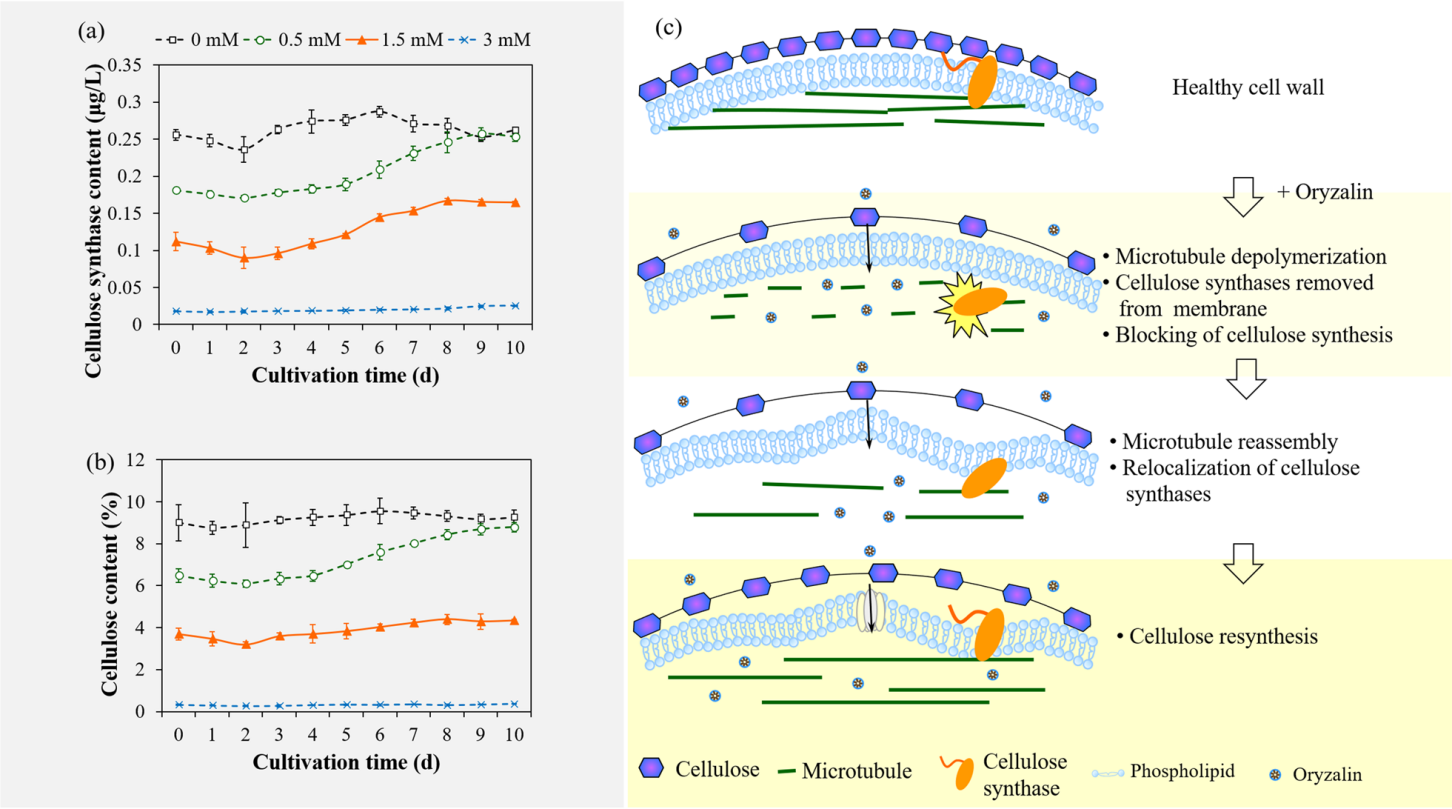
Effect of microtubules on cellulose synthase and cellulose synthesis in *Chlorella sorokiniana* SDEC-18: **a** cellulose synthase content of microalgae pretreated with 0 mM, 0.5 mM, 1.5 mM and 3 mM of oryzalin as a function of cultivation time; **b** cellulose content as a function of cultivation time; and **c** schematic diagram illustrating the mechanism through which microtubules affect cellulose synthesis

The temporal profiles of cellulose synthase and cellulose content in *Chlorella sorokiniana* SDEC-18 pretreated with 0, 0.5, 1.5 or 3 mM oryzalin, are presented in Fig. 4a, b. In microalgae pretreated with 0 mM oryzalin, the cellulose synthase and cellulose content tended to be high during the entire cultivation period. In comparison, the cellulose synthase and cellulose content in microalgae pretreated with oryzalin were clearly reduced, especially for 1.5 and 3 mM, which were depressed to just 0.16 and 0.03 μg/L for cellulose synthase content and 4.34 and 0.36 μg/L for cellulose content, respectively. For *Chlorella sorokiniana* SDEC-18 pretreated with 0.5 mM oryzalin, because the microtubules were able to be reassembled, the cellulose synthase and cellulose content were resynthesized accordingly, nearly reaching the levels in microalgae without pretreatment (0 mM).

Viewing all of this information together, there must be inextricable links between microtubules, cellulose synthase, and cellulose. As depicted in Fig. 4c, cellulose synthase was guided and immobilized on the cell membrane by microtubules, and cellulose synthase is able to affect the synthesis of cellulose [19]. When microtubules were depolymerized, the microtubule-guided cellulose synthase was removed from the membrane, which further affected the synthesis of cellulose. If the microtubule destruction were tempered (as in pretreatment with 0.5 or 1.5 mM oryzalin), the microtubules would be able to be reassembled, and microtubule-guided cellulose synthase would be relocalized on the cell membrane, and then the cellulose could be resynthesized [19]. But when the microtubule destruction is severe (as in pretreatment with 3 mM oryzalin), which were not stable, and the cellulose synthase cannot return to the cell membrane to fill the plasma membrane, causing morphological defects of algal cells [19].

#### Mechanisms of lipid accumulation and extraction enhancement in microalgae

**Fig. 5:**
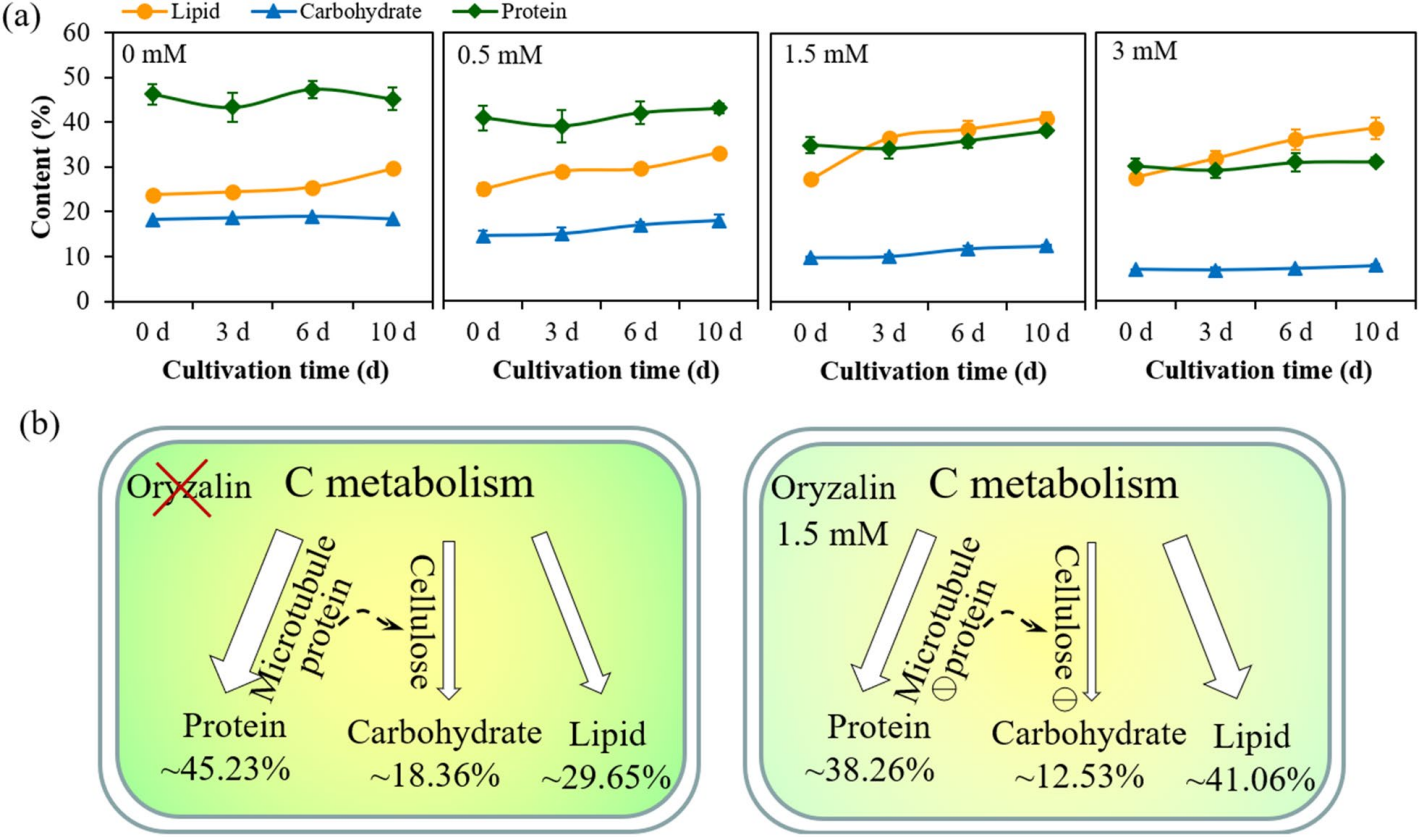
Mechanism of lipid synthesis within *Chlorella sorokiniana* SDEC-18 subject to microtubule disruption: **a** lipid, carbohydrate and protein content as functions of cultivation time; and **b** schematic diagram illustrating the mechanism of lipid synthesis in microalgae subject to microtubule disruption. indicate decreased activity

As mentioned above, an enhancement in lipid content was recorded in microalgal cells pretreated with oryzalin as compared to microalgal cells containing complete microtubules. As well as that, the time points of changes in lipid, microtubule protein, and cellulose were strikingly consistent through the aforedescribed real-time monitoring and observation. Therefore, it was probable that the microtubules promoted the lipid accumulation in microalgae by regulating carbon allocation in microalgal cells. Thus, we tracked total carbohydrate, protein and lipid content in microalgae pretreated with different concentrations of oryzalin (Fig. 5a). Compared to the microalgae without pretreatment, lipid accumulation was generally promoted while carbohydrate and protein content were reduced in microalgae pretreated with oryzalin.

The industrial demand of microalgae mainly depends on the intracellular biochemical components, such as lipids, carbohydrates, and proteins, which are closely related to external environmental factors because they can affect the carbon allocation in microalgae cells [45, 46]. As shown in Fig. 5b, the microtubule proteins in microalgae were destroyed, leading to the protein content being depressed from 45.23% down to 38.26%; furthermore, a decrease in the microtubule-affected cellulose content led to a decrease in carbohydrate from 18.36% to 12.53%. Therefore, in microalgae pretreated with oryzalin, more photosynthetic flow of carbon and energy from protein and carbohydrate was transferred into the biosynthesis of lipid/TAG than occurred in microalgae without pretreatment.

As for lipid extraction, when microtubules were depolymerized, the microtubule-guided cellulose synthase was removed from the membrane, which further affected the synthesis of cellulose to make cells fragile and thus easier to break.

### Using two-stage cultivation to enhance lipid productivity and extraction efficiency

#### Adverse impact of microtubule destruction on microalgal growth and lipid accumulation

**Fig. 6:**
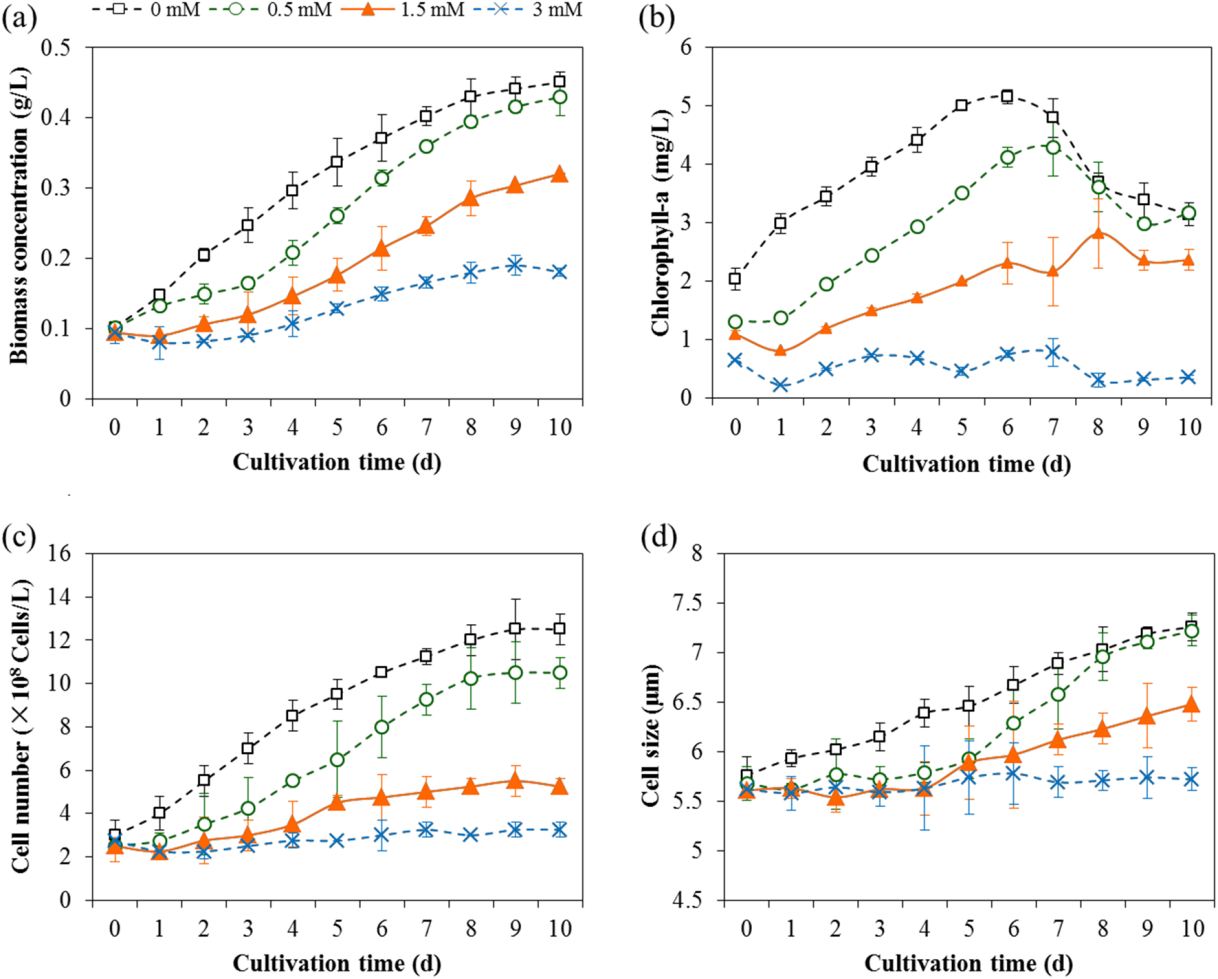
Effect of microtubules on the growth of *Chlorella sorokiniana* SDEC-18: **a** biomass concentration; **b** chlorophyll-a; **c** microalgal cell number; and **d** microalgal cell size, each as a function of cultivation time, for microalgae pretreated with 0 mM, 0.5 mM, 1.5 mM and 3 mM of oryzalin

The studies on microalgae with microtubule disruption suggested that microalgae efficiently modulated their metabolism in order to acclimatize themselves to the unfavorable circumstance of microtubule destruction. In such instances, microtubule destruction induced carbon redistribution: more carbon flow shifted from protein and carbohydrates to TAG, which yielded a large accumulation of lipid. Nonetheless, a retardation in biomass concentration and chlorophyll-a concentration were recorded in microalgal cells pretreated with oryzalin, as compared to microalgal cells containing complete microtubules (Fig. 6a, b). That was because microtubules could regulate the algal behaviors, such as cell division, proliferation and elongation [5, 6, 47], leading to a decrease in cell number and cell size under the unfavorable conditions of microtubule destruction (Fig. 6c, d) which resulted in inefficient lipid productivity (Fig. 1d). Therefore, there is still a long way to go to achieve the scales of algal biodiesel production based on present lipid yields.

#### The benefits of two-stage cultivation

**Fig. 7:**
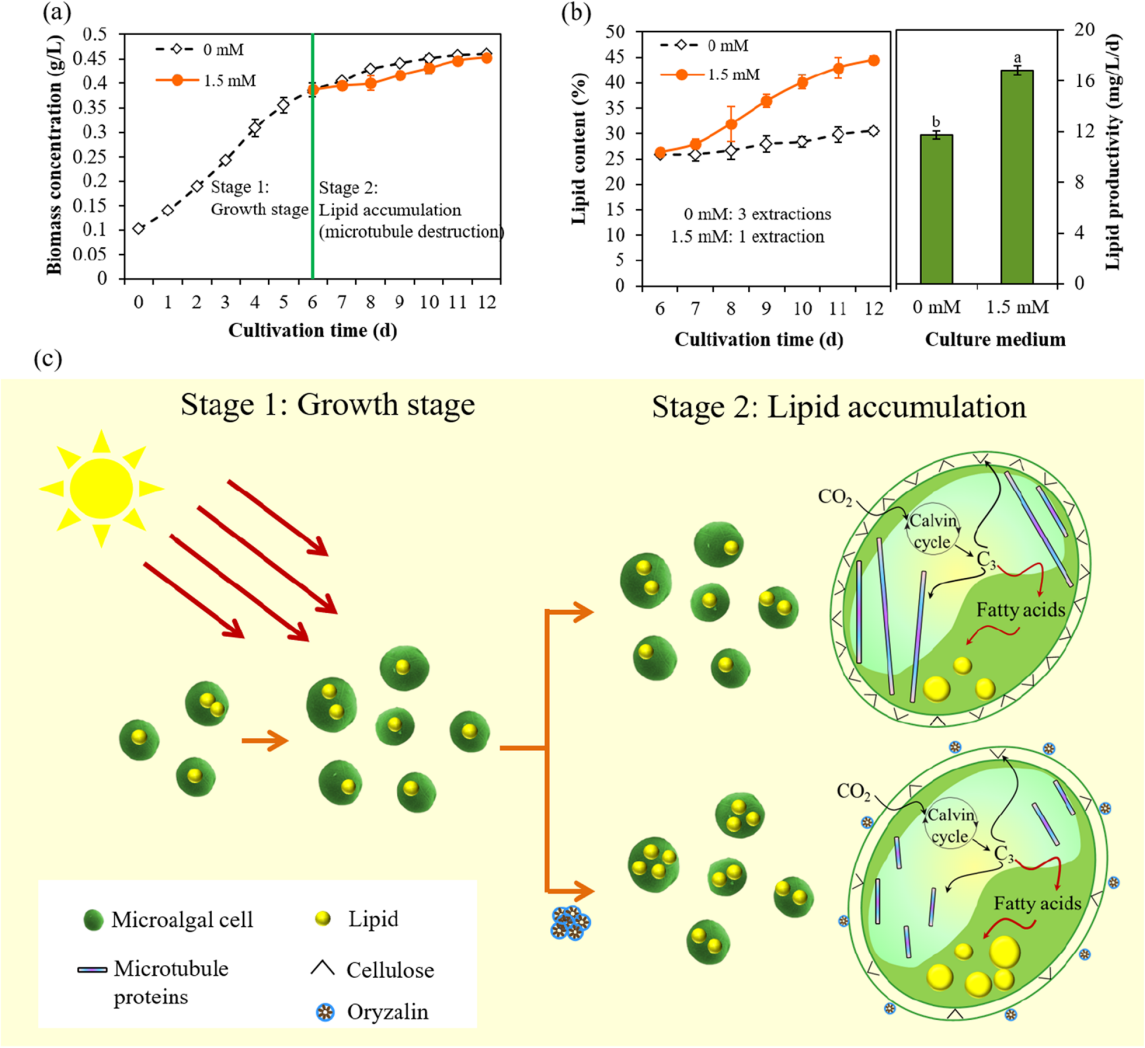
Growth and lipid accumulation of *Chlorella sorokiniana* SDEC-18 in two-stage cultivation involving microtubule destruction (1.5 mM oryzalin): **a** biomass concentration of microalgae in two-stage cultivation; **b** lipid content during the lipid accumulation stage from the 6th day to the 12th day, and lipid productivity; and **c** a schematic diagram of the two-stage cultivation of microalgae using oryzalin. Results not annotated with the same letter demonstrated a statistically significant difference (*p* < 0.05) between the corresponding treatments

Sparked by the inspiration of microalgae capable of accumulating high amounts of lipids following microtubule destruction, we proposed two-stage cultivation, in which microalgae grew ideally at first, and then the microtubules were destroyed to accumulate lipids when the microalgae entered the stationary phase. With regard to lipid accumulation, 1.5 mM oryzalin is a top priority in consideration of the lipid content and yields. As shown in Fig. 7a, a rapid increase in biomass concentration was recorded in microalgal cells during the growth stage (Day 0 to Day 6), and it remained constant thereafter. Strikingly, microalgae in two-stage cultivation involving microtubule destruction were able to reach the same growth level attained in normal BG11 medium. When microalgae entered the stationary phase, the lipid droplets started to be hyperaccumulated from the 7th day, reaching a content of 44.57% on the final day. Noteworthily, the lipid productivity of SDEC-18 in two-stage cultivation involving microtubule destruction—namely 16.85 mg/L/d—was 1.44 times higher than that in untreated microalgae (0 mM) (Fig. 7b). That is because microalgae accumulated sufficient biomass and lipid, in turn, during the two-stage cultivation involving oryzalin pretreatment (Fig. 7c). Also, in two-stage cultivation, the lipid-extraction efficiency was promoted: nearly complete extraction of the lipid required 3 successive extractions for microalgae grown in normal BG11 medium. In contrast, complete extraction was achieved after only one extraction step in microalgae harvested from two-stage cultivation involving microtubule destruction (Fig. 7b).

#### Fatty acid compositional profiles and biodiesel properties

**Fig. 8:**
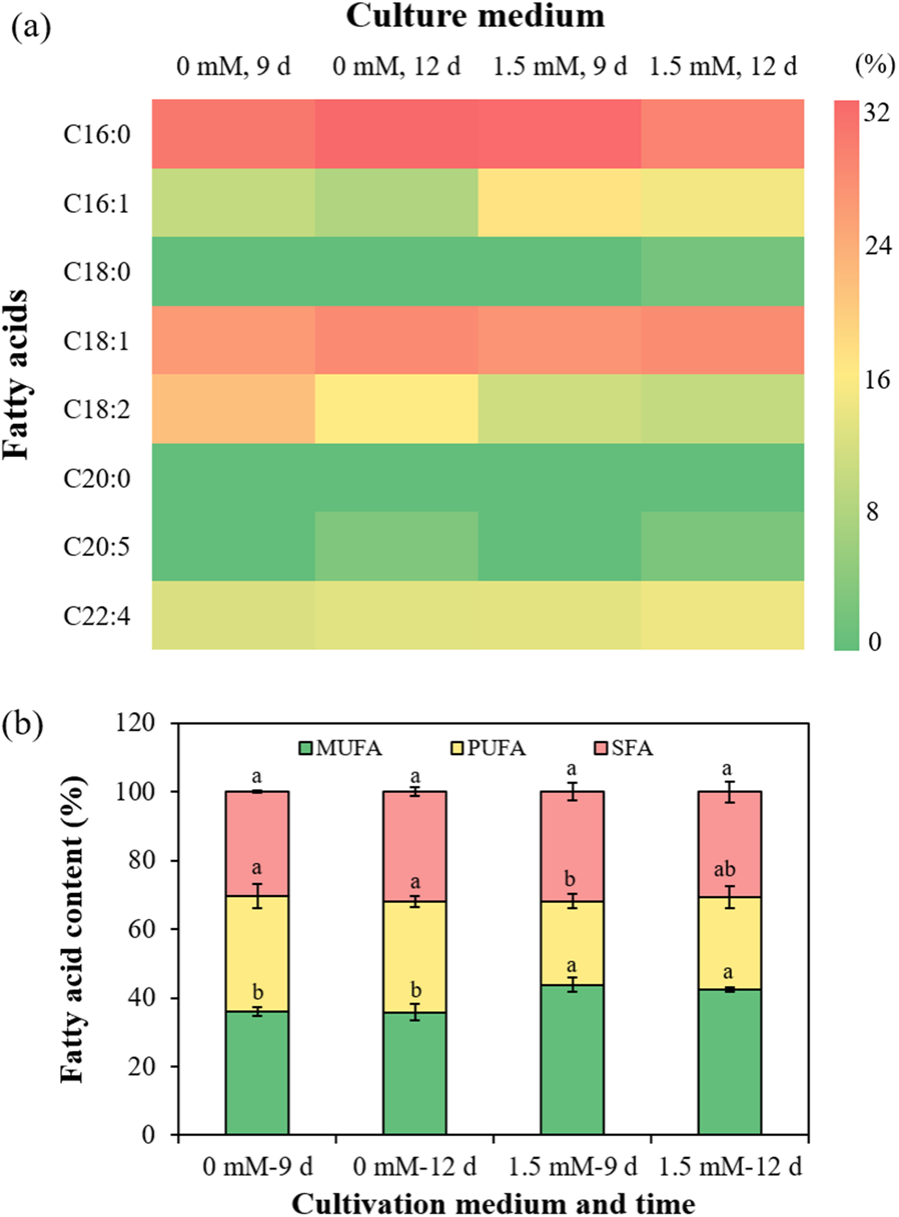
Fatty acid compositional profiles of *Chlorella sorokiniana* SDEC-18 in two-stage cultivation involving microtubule destruction (1.5 mM oryzalin): **a** fatty acids; and **b** the proportions of monounsaturated fatty acids (MUFA), polyunsaturated fatty acids (PUFA), and saturated fatty acids (SFA) in the fatty acids as a whole

Fatty acid profiles are a top priority in considering the quality of microalga-based biodiesel, and the fatty acid compositional profiles of *Chlorella sorokiniana* SDEC-18 are displayed in Fig. 8a. C16–C18 as the main class of components accounted for more than 80% of the fatty acids, and palmitic acid (C16:0) and oleic acid (C18:1) were the main components. Furthermore, an increase in monounsaturated fatty acid (MUFA) content was recorded in microalgae in two-stage cultivation involving microtubule destruction (Fig. 8b). The increase of monounsaturated fatty acids is mainly to maintain membrane fluidity of algal cells under adverse conditions [21, 48], which further indicates that *Chlorella sorokiniana* SDEC-18 was able to initiate a self-protective system and adapt to the stressful environment of microtubule destruction. Noteworthily, the proportion of C18:2 is decreased in untreated microalgae as the culture period increased from 9 to 12 days, while the proportion of C18:2 is relatively unchanged in 1.5 mM oryzalin-treated microalgae. That was because in fatty acids biosynthesis, C18:2 is more likely to be desaturated and elongated to C20, and further desaturated and elongated to C22, thus the contents of C20:5 and C22:4 increased from 9 to 12 days in microalgae. While in 1.5 mM oryzalin-treated microalgae, the conversion from C16:0 to C18:2 was catalyzed by Δ6-desaturase, and the activity of Δ6-desaturase might be enhanced by the uncomfortable environment [49]. Therefore, the proportion of C18:2 is relatively unchanged in 1.5 mM oryzalin-treated microalgae, while the proportion of C18:2 is decreased in untreated microalgae.

As for biodiesel properties, fortunately, biodiesel of microalgae cultivated in 0 or 1.5 mM oryzalin all satisfied the quality standards of Europe (EN) and the American Society for Testing and Materials (ASTM) (Table 1). A suitable cetane number (CN) of 54.03 was recorded in biodiesel derived from cells in two-stage cultivation involving microtubule destruction, harvested on the final day. The iodine value (IV) obtained was lower than 120 g I_2_/100 g, making the biodiesel less susceptible to gum formation [50]. The cold filter plugging point (CFPP) of the biodiesel derived from microalgae in two-stage cultivation involving microtubule destruction was about − 15 °C, suggesting its usage at low temperatures. CFPP is directly dependent on the content of saturated fatty acids (SFA), and in particular C16:0 and C18:0, as those two fatty acids precipitate faster at low temperatures [51]. The biodiesel derived from these cells showed a higher heating value (HHV) of 40.87 MJ/kg, kinematic viscosity (KV) of 4.37 mm^2^/s, cloud point (CP) of 2.27 °C, and density of 0.88 kg/L, which were all within the set range of acceptable standards for biodiesel.

**Table 1:** Biodiesel properties of *Chlorella sorokiniana* SDEC-18 after two-stage cultivation involving microtubule destruction

Biodiesel property	0 mM, 9 d	0 mM, 12 d	1.5 mM, 9 d	1.5 mM, 12 d	EN	ASTM
CN	54.34	53.85	54.94	54.03	> 51	> 47
IV (gI_2_/100 g)	107.91	113.42	101.24	111.38	< 120	–
CFPP (°C)	− 15.03	− 14.95	− 15.10	− 14.96	–	–
CP (°C)	2.90	1.91	3.10	2.27	–	–
KV 40 °C (mm^2^s^−1^)	4.40	4.35	4.45	4.37	3.5–5.0	1.9–6.0
Density (kg/L)	0.88	0.88	0.88	0.88	0.86–0.90	–
HHV (MJ/kg)	40.79	40.92	40.63	40.87	–	–

– No limit provided

In summary, the technology of microtubule destruction was able to not only promote lipid accumulation, but also allow production of biodiesel with desirable properties. Moreover, two-stage cultivation involving microtubule destruction achieved the coupling of efficient algal growth, efficient accumulation of lipids with desirable properties, and efficient lipid extraction, which paves the way for commercialization of algal oils and advances our knowledge in both the applied and fundamental research areas of algal biodiesel production.

## Conclusion

Microtubule destruction, through pretreatment with different concentrations of oryzalin, induced carbon redistribution: more photosynthetic flow of carbon and energy from protein and carbohydrate was transferred into the biosynthesis of lipid/TAG, which achieved a large accumulation of lipid. Limited by the growth inhibition of microalgae with microtubule destruction, we proposed two-stage cultivation involving microtubule destruction. In that case, the lipid productivity of *Chlorella sorokiniana* SDEC-18 reached 16.85 mg/L/d, being 1.44 times higher than that for microalgae without pretreatment; and complete extraction of lipid was achieved after only a single extraction step, while microalgae without pretreatment required 3 successive extractions for near-complete extraction of the lipid. Additionally, the microalgae in two-stage cultivation involving microtubule destruction were able to gain desirable properties for production of an ideal biodiesel. Therefore, two-stage cultivation involving microtubule destruction achieved the coupling of efficient algal growth, efficient accumulation of lipid with desirable properties and efficient lipid extraction, forging a new path for economically viable lipid-induction strategies in microalgae to accelerate the application of algal biofuels as environmentally sustainable alternatives to fossil fuels.

## Supplementary Information

**Additional file 1: Fig. S1.** Schematic diagrams showing the use of oryzalin to depolymerize microtubules of algal cells. **a** Using oryzalin to pretreat microalgae cells. **b** Two-stage cultivation of microalgae using oryzalin. **Fig. S2.** Transmission electron micrographs of microalgae cells, with orange arrows indicating lipid droplets, green arrows indicating starch grains. **Fig. S3.** Transmission electron micrographs of colloidal gold-labeled microtubules in the lipid accumulation stage, in which black particles are colloidal gold particles indicating the presence of microtubules, while green arrows show separation of the cell plasma and wall.

## Data Availability

All data generated and analyzed in this study are included in this published article.
