# Supplementary material for: The role of microtubules in microalgae: promotion of lipid accumulation and extraction
Source: Biotechnol Biofuels Bioprod. 2023 Jan 12;16:7. doi: 10.1186/s13068-023-02257-8 (PMC9837904; doi:10.1186/s13068-023-02257-8)
Supplement: Supplementary file 1 — Additional file 1: Fig. S1. Schematic diagrams showing the use of oryzalin to depolymerize microtubules of algal cells. a Using oryzalin to pretreat microalgae cells. b Two-stage cultivation of microalgae using oryzalin. Fig. S2. Transmission electron micrographs of microalgae cells, with orange arrows indicating lipid droplets, green arrows indicating starch grains. Fig. S3. Transmission electron micrographs of colloidal gold-labeled microtubules in the lipid accumulation stage, in which black particles are colloidal gold particles indicating the presence of microtubules, while green arrows show separation of the cell plasma and wall. [file 13068_2023_2257_MOESM1_ESM.docx]

Additional file 1: Figures


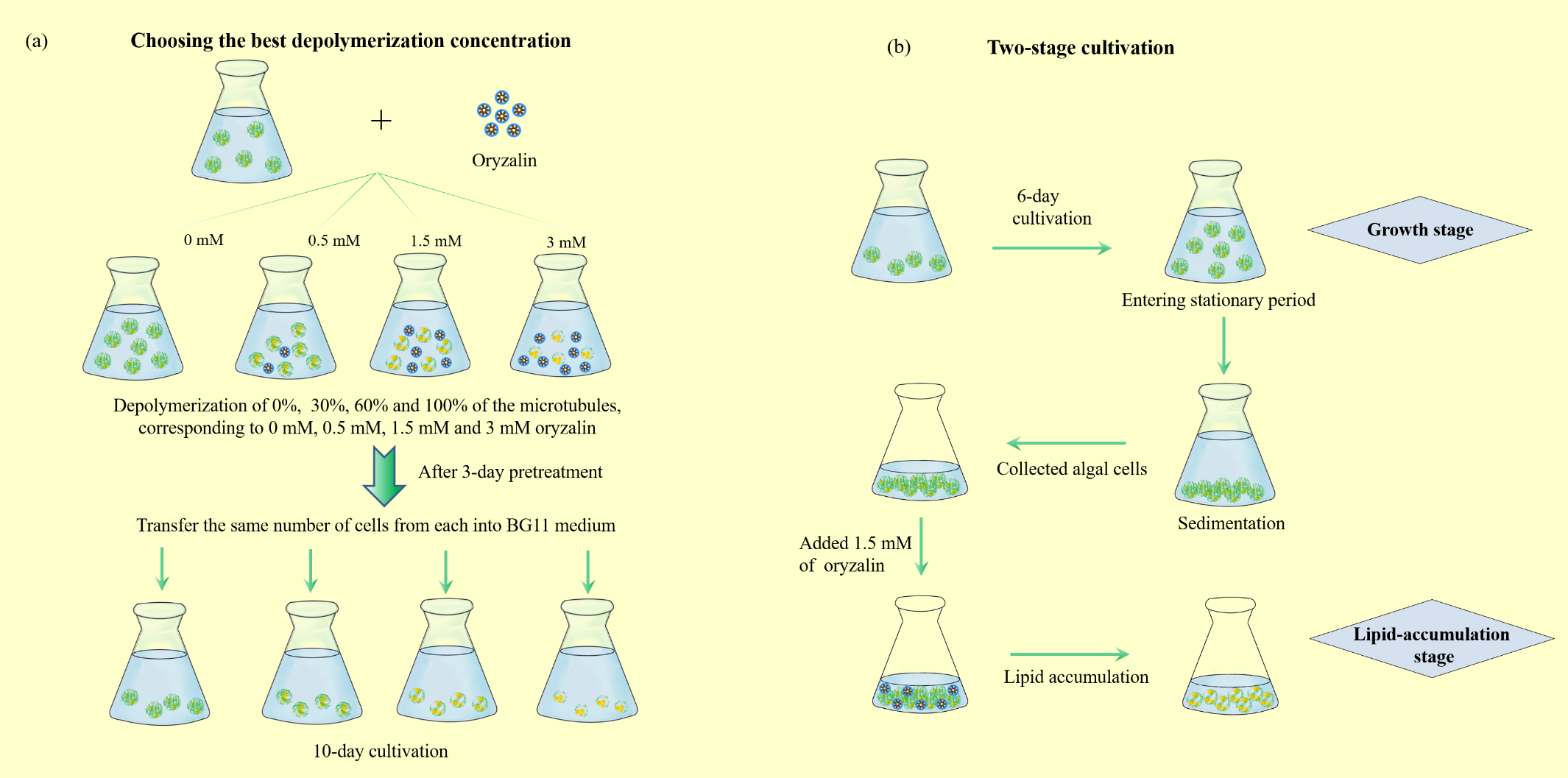


**Fig. S1.** Schematic diagrams showing the use of oryzalin to depolymerize microtubules of algal cells. (a) Using oryzalin to pretreat microalgae cells. (b) Two-stage cultivation of microalgae using oryzalin.


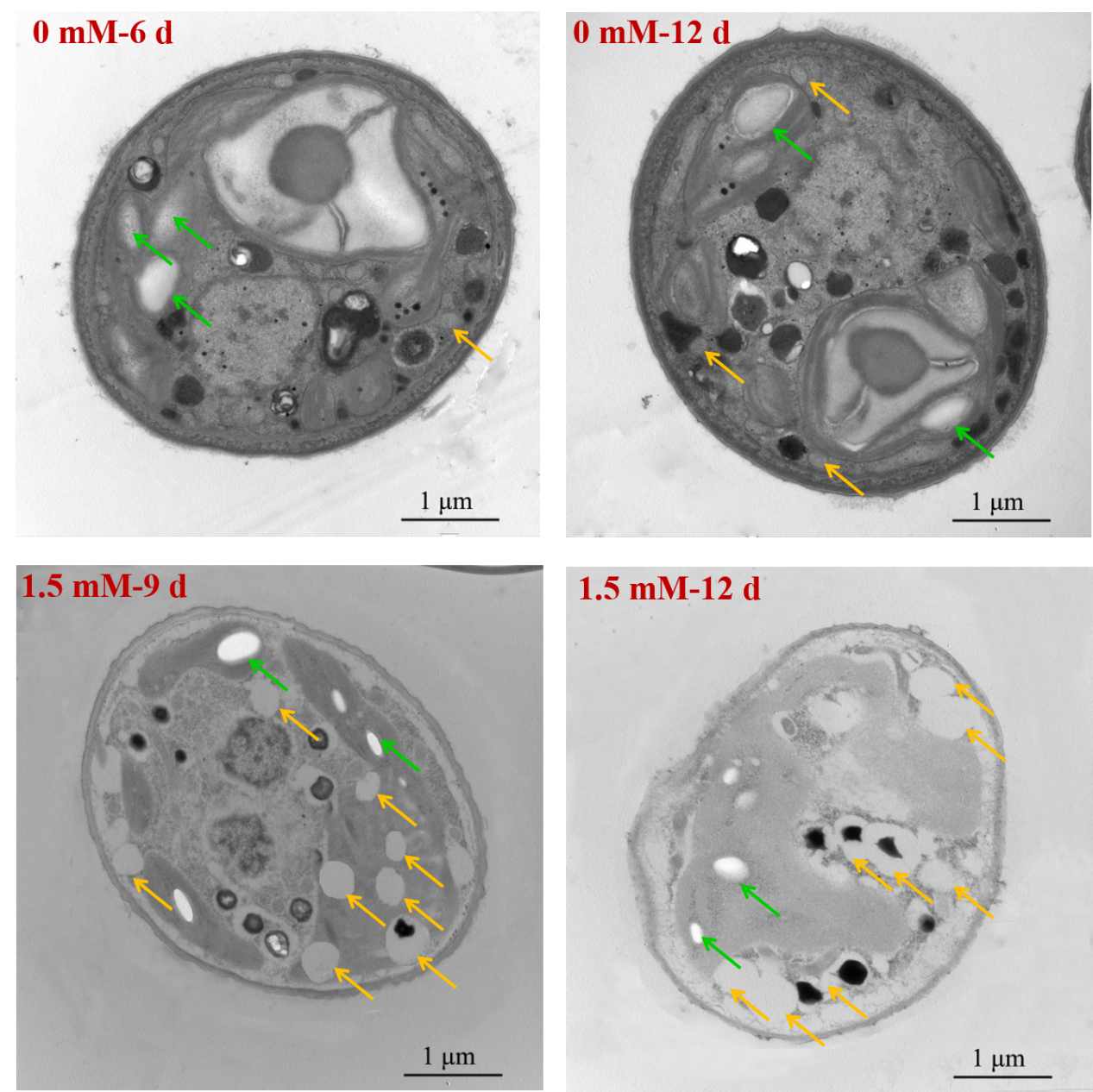


**Fig. S2.** Transmission electron micrographs of microalgae cells, with orange arrows indicating lipid droplets, green arrows indicating starch grains.


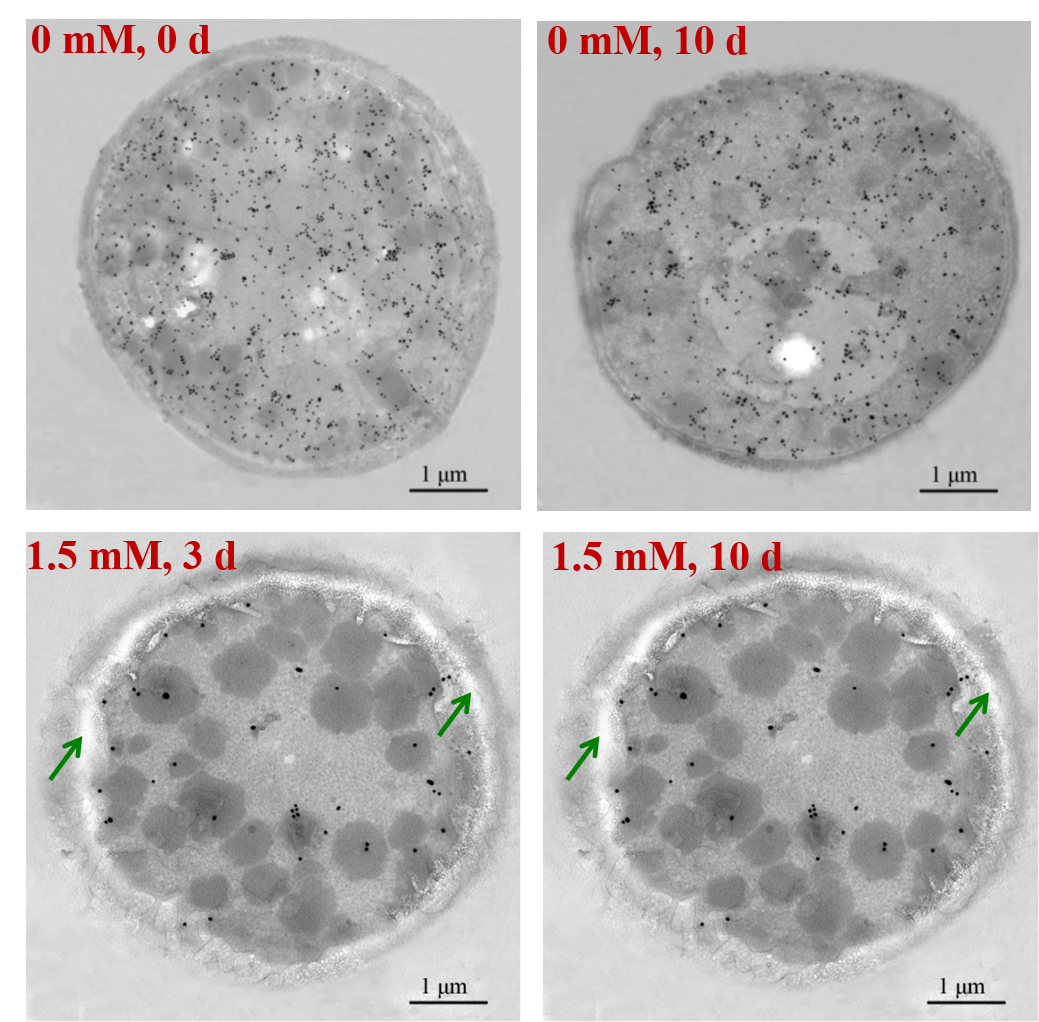


**Fig. S3.** Transmission electron micrographs of colloidal gold-labelled microtubules in the lipid accumulation stage, in which black particles are colloidal gold particles indicating the presence of microtubules, while green arrows show separation of the cell plasma and wall.
